# Continuous infusion of meropenem–vaborbactam for a KPC-3-producing *Klebsiella pneumoniae* bloodstream infection in a critically ill patient with augmented renal clearance

**DOI:** 10.1007/s15010-023-02055-2

**Published:** 2023-06-06

**Authors:** Romaric Larcher, Paul Laffont-Lozes, Tayma Naciri, Pierre-Marie Bourgeois, Cléa Gandon, Chloé Magnan, Alix Pantel, Albert Sotto

**Affiliations:** 1 Department of Infectious and Tropical Diseases, PhyMedExp (Physiology and Experimental Medicine), INSERM (French Institute of Health and Medical Research), CNRS (French National Centre for Scientific Research), University of Montpellier, Nimes University Hospital, Nimes, France; 2 Department of Infectious and Tropical Diseases, Nimes University Hospital, Nimes, France; 3 Department of Anesthesiology and Critical Care Medicine, Nimes University Hospital, Nimes, France; 4 Department of Microbiology and Hospital Hygiene, VBIC (Bacterial Virulence and Chronic Infection), INSERM (French Institute of Health and Medical Research), Montpellier University, Nimes University Hospital, Nimes, France; 5 Department of Infectious and Tropical Diseases, VBIC (Bacterial Virulence and Chronic Infection), INSERM (French Institute of Health and Medical Research), Montpellier University, Nimes University Hospital, Nimes, France; 6 Department of Pharmacy, Nimes University Hospital, Nimes, France; 7 Service Des Maladies Infectieuses Et Tropicales, Hôpital Caremeau—Centre Hospitalo-Universitaire de Nîmes, 1 Place Robert Debre, 30000 Nîmes, France

**Keywords:** Meropenem–vaborbactam, Continuous infusion, Augmented renal clearance, KPC-3-producing *Klebsiella pneumoniae*, ST11, ST147

## Abstract

**Purpose:**

To demonstrate the feasibility of continuous infusion of meropenem–vaborbactam to optimize the treatment of carbapenem-resistant Enterobacterales.

**Methods:**

Report of a case of a *Klebsiella pneumoniae* carbapenemase (KPC)-producing *K. pneumoniae* bloodstream infection comfirmed by whole genome sequencing and therapeutic drug monitoring (TDM) of meropenem.

**Results:**

A patient with augmented renal clearance (ARC) went into septic shock caused by an ST11 KPC-3-producing *K. pneumoniae* bloodstream infection that was successfully treated with a continuous infusion of meropenem–vaborbactam at a dosage of 1 g/1 g q4h as a 4-h infusion. TDM confirmed sustained concentrations of meropenem ranging from 8 to 16 mg/L throughout the dosing interval.

**Conclusion:**

Continuous infusion of meropenem–vaborbactam was feasible. It could be appropriate for optimizing the management of critically ill patients with ARC, as it resulted in antibiotic concentrations above the minimum inhibitory concentration for susceptible carbapenem-resistant Enterobacterales (up to 8 mg/L) throughout the dosing interval.

## Introduction

Antimicrobial resistance is increasing globally, resulting in longer hospital stays, higher medical costs, and increased mortality [1]. Fortunately, novel β-lactam/β-lactamase inhibitor combinations have been designed to target carbapenemase-producing Enterobacterales, which are among the most threatening resistant bacteria [1, 2].

Meropenem–vaborbactam is one of these novel antimicrobials, combining a carbapenem and a cyclic boronic acid β-lactamase inhibitor (inhibiting Ambler class A and C β-lactamases) with potent activity against *Klebsiella pneumoniae* carbapenemase (KPC)-producing Enterobacterales [2]. It was first approved by the US Food and Drug Administration (FDA) in 2017 with an optimized dosing regimen of 2 g/2 g (2 g of meropenem + 2 g of vaborbactam) q8h as a 3-h infusion to treat pathogens with MIC ≤ 8 mg/L [2, 3]. It is worth noting that dosage adjustment is recommended in patients with impaired renal clearance (< 50 mL/min/1.73 m^2^); however, there is no recommendation in patients with augmented renal clearance (ARC) [2–4]. For the latter, and in general for critically ill patients infected with carbapenem-resistant Enterobacterales, the use of continuous infusion (CI) could be interesting to limit therapeutic failures [5]. Indeed, like other β-lactams, meropenem–vaborbactam displays a predominantly time-dependent killing [2, 3]. Therefore, CI appears to be a promising dosing strategy to increase the likelihood of achieving the most optimized pharmacokinetic/pharmacodynamic (PK/PD) target [4, 8].

Here, we aimed to report the feasibility of meropenem–vaborbactam CI and to describe the meropenem pharmacokinetics in a patient with ARC and KPC-3-producing *K. pneumoniae* bloodstream infection (BSI) treated with this novel antimicrobial.

## Materials and methods

### Study design and setting

We reported the case of a patient hospitalized in Nimes University Hospital from 10/10/2022 to 9/12/2022, according to the CARE guidelines (for CAse REports).

### Augmented renal clearance

We defined ARC as a creatinine clearance (calculated from a 24 h urine collection) greater than 130 mL/min/1.73 m^2^ with a normal serum creatinine.

### Therapeutic drug monitoring

As meropenem and vaborbactam have shown comparable pharmacokinetic properties across varying degrees of renal dysfunction, therapeutic drug monitoring (TDM) of meropenem has been used as a surrogate for TDM of meropenem–vaborbactam [2]. Plasma concentrations of meropenem were determined using a high-performance liquid chromatography method [6].

### Microbiology

Minimum inhibitory concentrations (MICs) were determined with the Sensititre^™^ EUMDRXXF plate (ThermoFisher Scientific^™^, Waltham, MA, USA), except for the MICs of ertapenem which were determined by Etest^®^ (bioMérieux, Marcy-l’Etoile, France), and the MICs of cefiderocol which were determined with Liofilchem^®^ ComASP^®^ Cefiderocol (Liofilchem^®^, Roseto degli Abruzzi, TE, Italy) broth microdilution panel.

Carbapenemase was determined with the Xpert Carba-R test (Cepheid, USA, CA) in routine practice. Whole genome sequencing (WGS) was performed with the MiSeq System^®^ using the Nextera^®^ index kit (Illumina^®^, San Diego, CA, USA). Sequences were analysed by whole genome multilocus sequencing typing (wgMLST) with bioMérieux EPISEQ^®^ CS (V1.2) software (19729 *loci* analysed, including 1515 in the core genome). *K. pneumoniae* strains were considered epidemiologically linked when their genomes contained less than 23 allelic differences [7].

## Case presentation

In September 2022, a 46-year-old man, weighing 77 kg, with a medical history of Child C cirrhosis, was hospitalized for 3 weeks in Portugal, for variceal haemorrhage. In October 2022, he was admitted to a French tertiary hospital for ascites and jaundice. He was rapidly treated with ceftriaxone for 7 days for an undocumented spontaneous bacterial peritonitis. The rectal swab performed upon admission was positive for a KPC-producing *K. pneumoniae* (strain A, Table 1). Between day 7 and 27, hepatic encephalopathy and three episodes of acute variceal bleed occurred, requiring intensive care unit (ICU) admission, two endoscopic variceal ligations, a transjugular embolization of rectal varices, a transjugular intrahepatic portosystemic shunt, and an additional course of ceftriaxone for 10 days.

**Table 1:** Minimum inhibitory concentrations (MICs) of the main last-resort antibiotics against bacterial isolates from rectal swab and blood cultures of a critically ill cirrhotic patient

Antibiotic	Minimum inhibitory concentrations (mg/L)			2022 EUCAST breakpoints (mg/L)
	KPC-3-producing *K. pneumoniae* in rectal swab (strain A)	CTX-M-15-producing *K. pneumoniae* in blood cultures (strain B)	KPC-3-producing *K. pneumoniae* in blood cultures (strain C)	
Ertapenem	16^a^	0.125^a^	6^a^	0.5
Imipenem	4^b^	< 1^b^	8^b^	2–4
Meropenem	2^b^	< 0.12^b^	8^b^	2–8
Ceftolozane–tazobactam	> 8^b^	2^b^	> 8^b^	2
Ceftazidime–avibactam	2^b^	0.5^b^	1^b^	8
Imipenem–relebactam	0.12^b^	0.25^b^	0.5^b^	2
Meropenem–vaborbactam	< 0.06^b^	< 0.06^b^	< 0.06^b^	8
Cefiderocol	0.25^c^	0.5^c^	1^c^	2
Amikacin	< 2^b^	4^b^	4^b^	8
Colistin	< 0.5^b^	< 0.5^b^	< 0.5^b^	2
Tigecycline	< 0.5^b^	< 0.5^b^	< 0.5^b^	0.5
Eravacycline	0.25^b^	0.5^b^	0.5^b^	–

*KPC* *Klebsiella pneumoniae* carbapenemase, *ESBL* extended spectrum β-lactamase, *EUCAST* European Committee on Antimicrobial Susceptibility Testing. The type of carbapenemase was determined with the Xpert^®^ Carba-R test (Cepheid, Sunnyvale, CA, USA)

^a^MICs determined with Etest^®^ (bioMérieux, Marcy-l’Etoile, France)

^b^MICs determined with Sensititre^™^ EUMDRXXF panel (ThermoFisher Scientific^™^, Waltham, MA, USA)

^c^MICs determined with Liofilchem^®^ ComASP^®^ Cefiderocol (Liofilchem^®^, Roseto degli Abruzzi, TE, Italy) broth microdilution panel

**Fig. 1:**
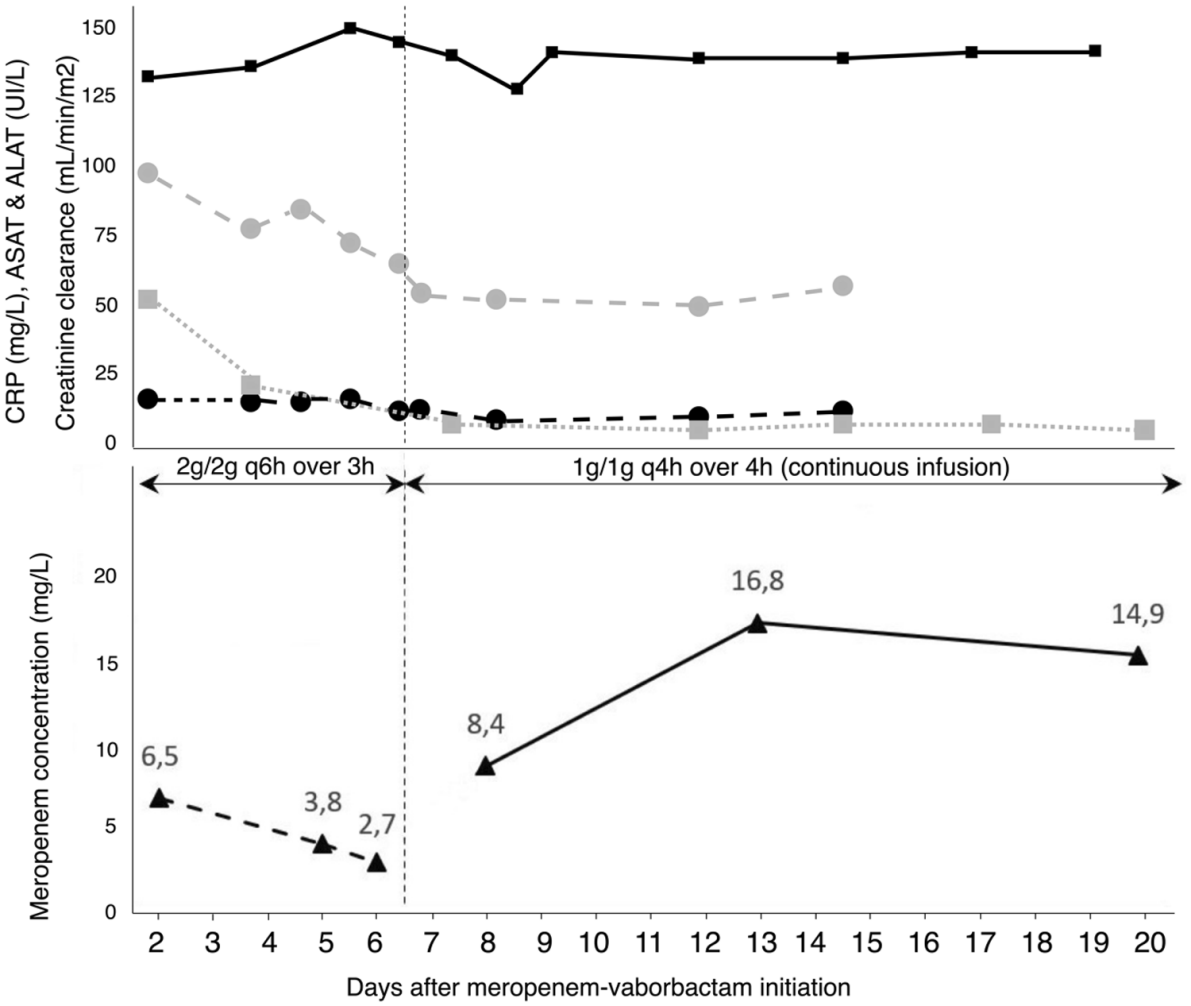
Therapeutic drug monitoring of plasma meropenem concentrations in a critically ill patient with augmented renal clearance (> 130 mL/min/1.73 m^2^) treated with meropenem–vaborbactam at 2 g/2 g q6h as a 3-h infusion (extended intermittent infusion of 8 g/8 g/day), then at 1 g/1 g q4h as a 4-h infusion (CI of 6 g/6 g/day). Meropenem trough concentrations are represented by triangles and a dashed black line, and steady-state concentrations by triangles and a black line. Creatinine clearance is represented by squares and a black line, alanine transaminase (ALT) and aspartate transaminase (AST) are represented by circles and dotted black and grey lines, respectively, and C-reactive protein (CRP) is represented by squares and a dotted line

On day 27, his condition worsened due to septic shock, empirically treated with meropenem and amikacin. The blood cultures, drawn for fever 1 day prior, were positive for an extended spectrum β-lactamase (ESBL)-producing *K. pneumoniae* (strain B, Table 1). On day 29, the patient was still febrile and in septic shock, and the antimicrobials were switched to ceftazidime–avibactam (2 g/500 mg bolus followed by 2 g/500 mg q8h as an 8-h infusion). On day 33, the blood cultures drawn on day 32 were positive for KPC-producing *K. pneumoniae* (strain C, Table 1). On day 34, as the patient still had fever and sepsis, antimicrobials were switched to the one with the lowest MIC. Meropenem–vaborbactam 2 g/2 g q6h as a 3-h infusion (8 g/8 g/day due to ARC at 140 mL/min/1.73 m^2^) was started and catheters were replaced. No septic focus was evidenced on computerized tomography (CT) scan and positron emission tomography (PET) scan. Meropenem through concentrations on days 36, 39 and 40 were 6.5 mg/L, 3.8 mg/L and 2.7 mg/L, respectively. On day 40, the dosing regimen was optimized to 1 g/1 g q4h as a 4-h infusion corresponding to a CI of 6 g/6 g/day. Meropenem steady-state concentrations were 8.4 mg/L, 16.8 mg/L and 14.9 mg/L, on days 42, 47 and 54, respectively (Fig. 1). Blood cultures drawn on day 35, 36 and 47 were negative and the patient was discharged to home at the end of treatment (day 61). There was no evidence of adverse effects, such as hypersensitivity reaction, neurotoxicity or acute kidney injury during treatment.

**Fig. 2:**
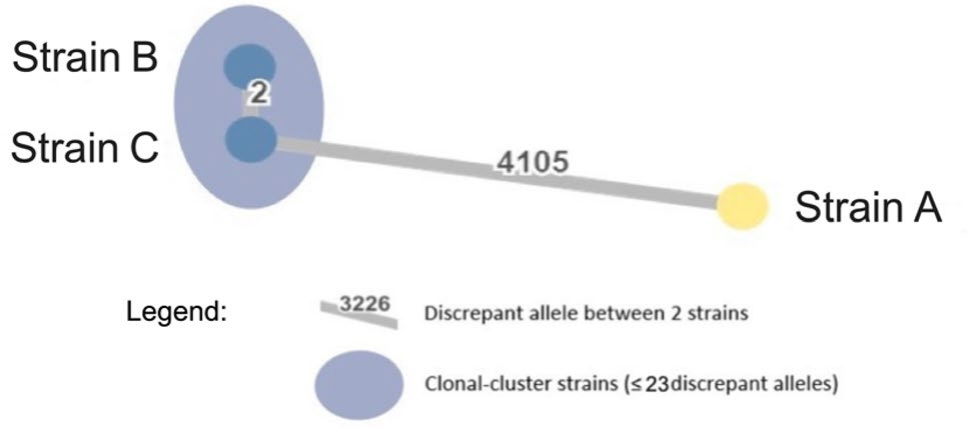
Minimum spanning (EPISEQ®CS) of allelic distances separating *Klebsiella pneumoniae* strains in stool (**A**) and blood cultures (**B** and **C**)

Interestingly, wgMLST showed that the KPC-3-producing *K. pneumoniae* strains A and C were not related (19.78% of sequence similarity) and belonged to international high-risk clones ST147 and ST11 (see Fig. 2 and Table 2). On the contrary, the KPC-3-producing *K. pneumoniae* strain C and the CTX-M-15-producing *K. pneumoniae* strain B isolated from the blood cultures were very similar (99.97%). They carried the same *traT* virulence gene on an IncFIB(K) plasmid. which encodes for an outer membrane protein of complement resistance and the same resistance genes on the IncFIA(HI1),IncR plasmid, except for the *bla*_KPC-3_ gene harboured by an IncN plasmid.

**Table 2:** Dendrogram, percentages of sequence similarity, resistome, virulomes and plasmids of different *Klebsiella pneumoniae* strains

Similarity percentage	Strain	Collection date	Specimen type	MLST	Resistance and virulence genes carried on plasmids					
					Beta-lactamase		Aminoglycosides inactivating enzymes	Cotrimoxazole modified target	Quinolone target-site protection	Resistance to serum
	C	11/11/2022	Blood	ST11	IncN	IncFIA(HI1), IncR				IncFIB(K)
					*bla*_KPC-3_	*bla*_CTX-M-15_, *bla*_OXA-1_	*aac(6′)-Ib-cr, aph(3′′)-Ib, aph(6)-Id*	*sul2, dfrA14*	*qnrB1*	*traT*
	B	05/11/2022	Blood	ST11		IncFIA(HI1), IncR				IncFIB(K)
						*bla*_CTX-M-15_, *bla*_OXA-1_	*aac(6′)-Ib-cr, aph(3′′)-Ib, aph(6)-Id*	*sul2, dfrA14*	*qnrB1*	*traT*
	A	11/10/2022	Rectal swab	ST147	IncN					
					*bla*_KPC-3_					

## Discussion

To our knowledge, this is the first report of meropenem–vaborbactam administration by CI. The body of evidence suggests that CI of ß-lactams may be of interest in ICU patients with normal or ARC, to improve pharmacokinetic-target attainment, coverage of less-susceptible pathogens, bactericidal activity and outcomes [4, 8]. In accordance, we reported that only CI of meropenem–vaborbactam resulted in sustained concentrations above the MIC of susceptible pathogens throughout the whole dosing interval (100%ƒT > _MIC_), taking into consideration the 8 mg/L breakpoint [2, 3]. There is no optimized pharmacokinetic target available in the literature for meropenem–vaborbactam. However, it is likely that the optimized targets for other beta-lactams, i.e. a free drug concentration above fourfold the MIC throughout the whole dosing interval (100%ƒT > _4xMIC_), can be applied [9]. We found that the CI of meropenem–vaborbactam achieved concentrations between 8 and 16 mg/L, thus reaching the optimized pharmacokinetic target of 100%ƒT > _4xMIC_, for MIC up to 4 mg/L [10]. In addition, vaborbactam exhibits concentration–time profiles, suggesting that a pharmacokinetic target higher than 100%ƒT > _4xMIC_ could enhance bactericidal activity in infections due to KPC-producing Enterobacterales [2]. Therefore, even at the lowest MICs, CI of meropenem–vaborbactam could be of interest.

The CTX-M-15-producing *K. pneumoniae* (strain B) and the KPC-3-producing *K. pneumoniae* (strain C), involved in the BSIs, shared the IncFIB(K) plasmid with the *traT* gene that encodes an outer membrane protein of complement resistance, allowing the bacteria to escape the host immune system [11]. WGS suggested that strain C probably picked up the plasmid IncFIA(HI1), IncR with the *bla*_KPC-3_ gene, frequently described in Portugal [12, 13], from the KPC-3-producing *K. pneumoniae* (strain A) stool carriage. This case emphasizes the ability of *K. pneumoniae* to get new plasmids from other bacteria, which increases its resistance and virulence, and should prompt physicians to optimize drug selection and dosage in most severely ill patients [14].

Importantly, we reported that CI of meropenem–vaborbactam was feasible and safe when the daily dose was divided into six 4-h infusions in agreement with the manufacturer’s recommendations that meropenem–vaborbactam should not be infused for longer than 4 h, which is its stability limit at room temperature [2, 3, 15].

This study has limitations. First, the results presented in this case report need to be confirmed by larger studies. However, to our knowledge, this is the first clinical reference in the literature for the use of meropenem–vaborbactam CI. Second, we did not perform TDM of vaborbactam. Nevertheless, its pharmacokinetics seems to be comparable to that of meropenem [2]. Finally, we reported the effectiveness of meropenem–vaborbactam CI for bacteria with an MIC of 0.06 mg/L, and the effectiveness for bacteria with MICs up to 8 mg/L needs to be confirmed.

## Conclusions

This case of successful microbiological and clinical treatment of a KPC-3-producing *K. pneumoniae* BSI in a critically ill patient with ARC, supports the use of meropenem–vaborbactam CI at a dosage of 1 g/1 g q4h as a 4-h infusion to achieve steady-state meropenem concentrations of 8–16 mg/L. Further investigations are warranted to confirm our results and to assess the effect of such a dosing regimen on the outcome of critically ill patients infected with carbapenem-resistant Enterobacterales.

## Data Availability

The authors consent to share the collected data with others. Data will made available by the authors, without undue reservation, immediately after the main publication and indefinitely.
